# Third Fasciculus of Anatomical Drawings, Selected from the Collection of Morbid Anatomy in the Army Medical Museum at Chatham

**Published:** 1839-01-01

**Authors:** 


THE THIRD FASCICULUS OF ANATOMICAL DRAWINGS, SELECTED FROM THE COLLECTION OF MORBID ANATOMY IN THE ARMY MEDICAL MUSEUM AT CHATHAM. Drawn on Stone by *Waterhouse Hawkins*. London, 1838.

THE Army Medical Museum at Chatham has become celebrated as a collection of morbid preparations. Many have visited, and all, who can, should visit it. The zeal displayed in its formation, and the care and talent exhibited in the display of the specimens, have won golden opinions from all competent judges.

But the anxiety of the Medical Department of the Army to encourage pathological science has not stopped at the construction of a museum. To render its stores as available as possible to medicine, drawings of the more remarkable preparations have been published, and the present is the Third Fasciculus of this valuable work.

We are informed in the Preface that, since the publication of the last Fasciculus, in 1834, the anatomical collections belonging to the museum have continued increasing in magnitude and value. The number of preparations at present is as follows:—Of Morbid Anatomy 2316—Of Healthy Anatomy 685—Of Comparative Anatomy 740—Of Experimental Physiology 100.

Government has granted additional room, and the reporters hope that the future progress of the museum will be even greater than the past. The reporters remark, that:—

Many instances are given of lesions, which were not suspected during life, and were yet doubtless of long standing. These and other analogous facts, are strongly indicative of the difficulties and imperfections of the diagnostic part of medicine; and how careful and vigilant the medical officer should be in examining men brought before him with obscure ailments, and vague complaints, lest he overlook serious organic disease, and unjustly condemn men of "malingering."

The imperfection of diagnosis referred to, may be considered as dependent on imperfection of observation, to be corrected only by increased attention, directed by precise knowledge, to doubtful points, and aided whenever available by improved methods of scrutiny. Thus, probably, many latent diseases may be detected; as disease of the investing cartilages of joints, by the sensation of roughness imparted to the touch of the examiner, as pointed out by Mr. Gulliver;* as disease of arteries, and probably aneurysms in very many cases, by loss of the pulse in some one or other of the larger arteries; not to mention obscure affections of the heart and arteries, for the detection of which we possess invaluable aids in auscultation and percussion.

By the results of researches, bringing to light the obscure and latent organic changes to which so many textures of the body are liable, pathological anatomy is intimately connected with practical medicine, and is brought to bear even on the inspection of recruits and invalids, imparting, it may be said, new power of discrimination to the examining officers. How invaluable then, in this point of view, as well as in innumerable others, is a museum, in which the results, as exemplified in the injured parts, are stored up and systematically arranged for examination and reference; and how deserving is it of the continued support of the department to which it belongs, and of the patronage of Government, merely in relation to its usefulness in connexion with the public service!

A few subjects selected from the division of Experimental Physiology have been included in this Fasciculus. This is a department in which, perhaps, much is yet to be done. We cannot join in the indiscriminate condemnation of what is calculated to give exactness to our view of the œconomy. Cruelty has no doubt been exercised, but that should be, and might be, in a great measure, avoided; and when millions are slaughtered to stimulate the sensualist's cloyed appetite, a few victims may surely be offered to science and the general interests of humanity.

We are glad to learn that:—

* Edinburgh Med. and Surg. Journal, Vol. xlviii.

"It may here be stated, that the Director General, having three years ago obtained the sanction of the Secretary at War, for making a digest from the valuable Records in the Office, of the History of the Diseases of the Army for the last twenty years, considerable progress has been made in this Work by the Officers employed on it, viz. Deputy Inspector General Marshall, Captain Tulloch, and Dr. Balfour, Staff Assistant Surgeon, and that Lord Howick has further ordered that the First Volume, which includes the West India Islands, be printed immediately. The Second Volume, which will include St. Helena, the Cape of Good Hope, and Ceylon, is likewise in progress; and Volumes will follow which give India, North America, the Mauritius, Western Africa, &c. Those valuable Records which give the Observations and Reports of the Medical Officers of the Army in every quarter of the World, where a British Soldier is stationed, are enriched by Statistical Statements of great interest by Captain Tulloch, extracted from the Reports and Returns in the Offices of the Army Medical Department and War Office."—*Pref.*

The Fasciculus before us contains ten lithographic plates, beautifully executed.

PLATE I. represents a species of organic disease of the liver, of an obscure kind, and as it is believed, not yet described; secondly, a bony growth in the substance of the organ; and thirdly, a communication through the diaphragm between an abscess in the liver and the lung.

The peculiar disease of the liver would seem to consist of numerous well-defined excavations, probably formed by the softening and breaking down of a peculiar tubercular matter. In the portions Nos. 1 and 2, there is both internally and externally a tubercular appearance. In No. 3 there is no such appearance, excepting partially in one cavity; the surface is smooth, and the internal substance apparently homogeneous.

PLATE II. represents first, the liver and gall-bladder, infested with lumbrici (ascaris lumbricoides); secondly, the gall-bladder, containing calculi; thirdly, the gall-bladder ulcerated; and, lastly, an enlarged biliary duct.

*Fig.* 1, represents the right lobe of the liver, the gall-bladder, and a portion of the duodenum of a Maltese boy, aged 2 years, who died with symptoms of dysentery. The stomach, duodenum, the gall-bladder, and biliary ducts contained lumbrici. The common duct and gall-bladder were greatly distended with them; and in some places they had penetrated through the ducts and the substance of the liver into the cavity of the abdomen. The trichocephalus dispars equally abounded in the cæcum.

PLATE III. represents perforations of the pleura, from ulcerations connected with tubercular excavations, commonly giving rise to pneumo-thorax.

*Fig.* 1. exhibits the left lung, with a portion of costal pleura. A small perforation is seen on the surface of the lung, near its apex. The whole extent of the pleura is lined with coagulated lymph, and the lung is very much compressed. From the body of P. Calmon, aged 28 years, who died of tubercular consumption, complicated with empyema and pneumo-thorax, the former following hydrothorax. He was sent home from Jamaica on account of phthisical symptoms and hæmoptysis after a fall. In the General Hospital at Fort Pitt pneumothorax suddenly occurred, consequent on a violent fit of coughing. The operation of tapping his chest was performed with a trochar attached to a flaccid bladder. The air thus collected was found by measure to be composed of—7 carbonic acid gas, 93 azote=100. Great relief followed the operation. About a month after a second operation was required for hydrothorax. Life was protracted about three months, with comparatively little suffering.

In a case in which the operation was not performed, the air contained in the right pleura was found by Dr. Davy to be composed of 13 carbonic acid gas—87 azote=100.

The operation was performed in another case. A large quantity of air escaped, and immediate relief followed. The patient lived in comparative ease for a fortnight.

Plate IV. represents, first, an instance of popliteal aneurysm, for which the usual operation failed of success, in consequence of free anastomoses between the arteria profunda and the femoral artery; secondly, true aneurysm accompanied with ossification; and, thirdly, a false aneurysm, in the sac of which there was a bony deposit in the clot.

*Fig.* 3, displays a small aneurysm at the commencement of the aorta containing a clot, in which is a distinct osseous deposit. The bony matter in the clot does not appear to have any connexion with the coats of the vessel. From an analysis made by Dr. Davy the bony deposit appears to consist of phosphate of lime, animal matter, a little carbonate of lime, and a trace of sulphate of lime.

*Fig.* 4, shews a popliteal aneurysm, for which the femoral artery was tied, and which became obliterated about four inches below the crural arch. Coarse injection has been thrown into the vessel, and has penetrated freely into the whole of the femoral and the posterior tibial arteries. The anastomosing branches connecting the vessel above and below the ligature, are of large size, particularly the perforating branches of the arteria profunda: the sac accordingly has not been diminished in size by the operation.

Plate V. represents obstructions and obliteration of arteries, connected with aneurysm.

In this plate we have, among others, instances of obliteration of the cavity of the arteria innominata and of the left carotid arteries at their origin—of obliteration of the arteria innominata only—and of obliteration of the left carotid only.

*Fig.* 3, exhibits an aneurysm of the arch of the aorta, with complete obliteration of the arteria innominata, and of the left carotid artery at their origin. The orifice of the left subclavian artery is diminished, but perfectly pervious. From G. Sellars, æt. 34, 38th Regiment, a man of dissolute habits, 18 years service in India. He was admitted into the General Hospital, at Fort Pitt, with anasarca of the lower limbs, and great difficulty of breathing; a sense of weight in the chest, with inability to lie down; which symptoms, he said, commenced four months previously, with pains at the root of the neck and betwixt the shoulders. He had been seven different times in hospital with rheumatism during the two years preceding his death. The pulse at the right wrist was not perceptible for the last two or three months of his life. He died suddenly on the eighth day after his admission into the General Hospital, without apparent suffering.

The aneurysm was found to have burst into the pericardium. The tumor contained a large fibrinous clot, and some of the veins proceeding to the superior cava were obliterated by pressure. Two pints and three-quarters of serous fluid were contained in the left pleura, one and three-quarters in the right, and a considerable quantity in the abdomen.

*Fig.* 4, shews an aneurysm of the arch of the aorta, with complete obliteration of the arteria innominata at its origin. There was no pulsation perceptible either in the right carotid or brachial artery for some time previous to his death; but in those of the left side the pulsation was natural. The patient died after protracted dyspnœa, without rupture of the aneurysm.

In another case of aneurysm of the arch of the aorta, occasioning complete obliteration of the left carotid, the patient died without rupture of the sac.

PLATE VI. represents, first, obliteration of arteries from thickening of inner coat; secondly, from the formation of a coagulum, unconnected with aneurysm; thirdly, a closure of a principal branch of the pulmonary artery, from the pressure of a small aneurysm of the aorta at its origin; fourthly, an ossified state of the ductus arteriosus in a child; and, lastly, different stages of a wounded artery in process of healing.

*Fig.* 1, exhibits complete obliteration of the left common iliac artery, and nearly perfect closure of the right internal iliac at its origin. The coats of the vessels are diseased, the outer thickened and condensed, the middle atrophied, the inner irregularly thickened and partially ossified. From a very robust man of the 95th Regiment, who died suddenly of pulmonary ecchymosis. The disease of the vessels produced no inconvenience during life, nor was it ever suspected. The coats of the aorta were more or less similarly affected.

*Fig.* 2, exhibits complete closure of the left carotid and subclavian arteries, apparently by coagulable lymph, to the extent of about an inch, beyond which they were not examined. The inner coat of the aorta is irregularly thickened in a slight degree, and presents several osseous deposits. The other coats are rather atrophied. From Hector M'Cullum, aged 36, a drummer of intemperate habits, twenty-two years in the service. A year previous to his death he became subject to dyspnœa, which gradually became very distressing. For the last two or three months no pulsation could be felt at either wrist, and during the last fortnight there was no pulsation of the left carotid, and very indistinct of the right. The arteria innominata was probably ultimately closed; this was not determined, the *post-mortem* examination having been conducted in a very hurried manner, owing to interruption.

The arch of the aorta was found slightly enlarged; the heart and its valves pretty natural: there were two pints of serum in the left pleura, and three in the right.

*Fig.* 5, The right pulmonary artery completely closed at its origin by the pressure of a small aneurysm arising from the concavity of the aorta, near to its base, the closure was permanent from adhesion. The inner coat of pulmonary artery contiguous had lost its natural smoothness. From Corporal Sherron, æt. 33, who died of pulmonary phthisis. The disease of the vessels was never suspected during life, the symptoms being only those of ordinary phthisis.

Plate VII. represents, first, hypertrophy and atrophy, with softening of the articular cartilages in instances of pulmonary consumption; secondly, of atrophy unconnected with this disease in the middle period of life; and, thirdly, of atrophy in old age.

The affections represented in this plate are interesting, but we must refer our readers to the plate itself.

Plate VIII. represents the effects of encephaloid disease in different bones of the same subject.

T. Ward, aged 24, 29th Regiment, had been long under treatment for chronic rheumatism and pains affecting different parts of the body. The symptoms appear to have been very indefinite and obscure. After many months suffering he became extremely emaciated and bed-ridden; and died with diarrhœa twelve months after his admission into the General Hospital. *Fig.* 1, shews the pelvis, which is very fragile and light, with many large excavations in the cancellous texture of its different bones. The left os pubis and part of the right ilium are completely destroyed. In the recent state the deficient parts were filled with encephaloid matter, which in some situations formed tumors. *Fig.* 2, exhibits two of the lumbar vertebræ. Their osseous tissue, like that of the pelvis, is very fragile, light, soft and expanded. When recent, the interstices were occupied by brain-like matter. *Fig* 3, a portion of one of the ribs. Its texture is much rarified, with numerous openings into the cancelli; bony laminæ have grown from its surface.

Plate IX. represents, first, fractures reunited after a definite time, illustrative of the process of reparation; secondly, membrane containing a deposit of bone, with the same view; thirdly, a fracture, with displacement of vertebræ, bearing on a practical point; and, lastly, fracture of the patella, the results of experiments on animals, in illustration of the peculiarities of the process of reparation in this lesion.

The only point we think it necessary to notice is shewn by *Fig.* 5, that of parietal bones, from which a large portion of the entire thickness has exfoliated. The deficiency is filled up by membranous matter; and there has been an attempt at reparation by the formation of small osseous plates in the membrane, quite detached from the old bone.

This drawing illustrates the formation of new bone at a distance from the old bone, a fact at variance with the opinion of Haller and Dethleef. The accidental callus in the rabbit's tibia is another example of new bone, formed quite independently of the vessels of the old bone.

From William Hall, 16th Light Dragoons, who had lost his palate bones and penis, from syphilitic disease of two years' duration. He had been several times salivated.

Plate X. represents, first, some of the leading phenomena of necrosis, the results of experiments on animals, by Mr. Gulliver; secondly, examples of necrosis from the human subject, exhibiting certain peculiarities, &c.

thirdly, the results of experiments showing the adhesion of dead to living bone, and that there is no absorption of the former when placed amongst living parts.

We shall not specifically allude to the figures in this plate, as we have fully noticed a paper on necrosis by Mr. Gulliver, in our present number.

Our readers may guess from the facts we have quoted the value of this Fasciculus, and the still greater value of the museum from which its drawings have been taken. We wish success to both.

